# Severity Stratification of Coronary Artery Disease Using Novel Inner Ellipse-Based Foveal Avascular Zone Biomarkers

**DOI:** 10.1167/iovs.65.12.15

**Published:** 2024-10-09

**Authors:** Natasa Jeremic, Maximilian Pawloff, Dmitrii Lachinov, Stephanie Rokitansky, Matthias Hasun, Franz Weidinger, Andreas Pollreisz, Hrvoje Bogunović, Ursula Schmidt-Erfurth

**Affiliations:** 1 Laboratory for Ophthalmic Image Analysis, Department of Ophthalmology and Optometry, Medical University of Vienna, Vienna, Austria; 2 Department of Ophthalmology and Optometry, Medical University of Vienna, Vienna, Austria; 3 Christian Doppler Laboratory for Artificial Intelligence in Retina, Department of Ophthalmology and Optometry, Medical University of Vienna, Vienna, Austria; 4 Department of Internal Medicine II, Division of Cardiology, Clinic Landstraße, Vienna, Austria

**Keywords:** oculomics, systemic disease, cardiovascular disease, OCT-A

## Abstract

**Purpose:**

Given the similarities between the retinal and coronary microvasculature, the retina holds promising potential to serve as a non-invasive screening tool for coronary artery disease (CAD). We aimed to develop novel inner ellipse-based metrics and discern whether foveal avascular zone (FAZ) alterations can serve as indicators for CAD presence and severity.

**Methods:**

Patients admitted to the Department of Cardiology who underwent coronary angiography were included. This resulted in an inclusion of 212 patients, of which 73 had no CAD. During the same visit, 6 × 6-mm (nominal size) fovea-centered optical coherence tomography angiography images of both eyes were acquired. The Gensini score (GS) was utilized to quantify CAD severity. Six known FAZ shape metrics were assessed and three novel biomarkers based on the inner ellipse were defined: absolute inner ellipse difference, Hausdorff distance, and Chamfer distance.

**Results:**

Eight out of nine metrics showed significant associations with the GS in the left eye. However, significant differences across three CAD severity groups were only demonstrated by the novel metrics. Utilizing the Chamfer distance, age, and sex, patients with and without CAD could be distinguished with an average area under the curve (AUC) of 0.89 (95% confidence interval [CI], 0.84–0.95). Moreover, three CAD severity groups could be discerned with a macro average AUC of 0.77 (95% CI, 0.72–0.84).

**Conclusions:**

A comprehensive assessment of FAZ shape descriptors was performed, and a strong association with CAD was found. The inner ellipse-based biomarkers especially demonstrated high predictive abilities for CAD presence and severity.

Cardiovascular disease (CVD) is a major global health challenge, with coronary artery disease (CAD) being a leading cause of death and a substantial socioeconomic burden. CAD is characterized by plaque formation and blockage of the epicardial coronary arteries.^1^ A discontinuity in the coronary blood flow can lead to myocardial infarction within the affected area.^2^ Lifestyle modification plays a crucial role in disease prevention and can help to reduce the incidence of severe cardiovascular events and its associated complications.^3^ Currently, the gold standard for the diagnosis and treatment of CAD is coronary angiography. Although effective, this approach is invasive and expensive and carries potential risks.^4^ Therefore, non-invasive diagnostic and screening tools would be beneficial for early detection and monitoring of the disease in the large epidemiological dimension needed.

As we explore more non-invasive diagnostic tools, the retinal microvasculature emerges as a promising area of interest. Given the analogies between retinal and coronary microvasculature, retinal microvascular changes have a huge potential to serve as subclinical CAD markers, as they are likely undergoing processes similar to those of cardiac microvessels.^5^^,^^6^ Aligning with these theoretical propositions, multiple studies have suggested the presence of detectable alterations in the retina of CAD patients.^7^^–^^9^

Optical coherence tomography angiography (OCT-A), a non-invasive, high-resolution imaging modality, enables the clinician to analyze retinal circulation using decorrelation signals. Utilizing this technology, circulatory abnormalities, such as loss of vessels allegedly caused by anatomical impairment of the vessels, become visible.^10^^,^^11^ The foveal avascular zone (FAZ) is a capillary-free region in the central macula circumscribed by a capillary network from branches of the central retinal artery. Irregularities and variations in the FAZ reflect both the central retinal and apparently the systemic circulation, as well.^10^^,^^11^ Despite the advancements offered by OCT-A, the significance of the FAZ in CAD has not been addressed heretofore. This may be largely due to focusing on inconsistent and non-specific metrics regarding the FAZ area.^8^^,^^12^^,^^13^ Although several structural FAZ metrics, such as circularity, solidity, or roundness, have been studied in the context of other diseases, no study examining their relationship with CAD has yet been performed.^10^^,^^14^^–^^18^ A further issue diminishing the diagnostic value of the FAZ so far is the lack of standardized annotation criteria, leading to inconsistent segmentation results, especially in diseased patients, who commonly present with very irregular FAZ borders.^11^^,^^19^^–^^21^

In an effort to harness the full potential of OCT-A imaging and to systematically explore the diagnostic value of the FAZ, we aimed to develop and test novel FAZ biomarkers for CAD that have not been previously assessed. Furthermore, we wanted to discern whether FAZ metrics can be used to identify CAD patients or even determine CAD severity.

## Methods

### Study Design and Workflow

We conducted a prospective cross-sectional study in patients with a comprehensive cardiological assessment. Ethical approval was obtained by the ethical committee of the Medical University of Vienna (EK: 1956/2020). All examinations and analyses were performed in accordance with the tenets of the Declaration of Helsinki. We included patients admitted to the Department of Cardiology at the Clinic Landstraße who underwent coronary angiography. Prior to inclusion, informed consent was obtained from all subjects. Both eyes of each patient were imaged, and one image per eye was considered for analysis. We excluded eyes of patients that provided a history of retinal disease or surgery and where excellent quality images were not obtainable (Supplementary Table S2). Moreover, patients with previous coronary stenting were excluded. Throughout their hospitalization, all patients received a comprehensive physical examination, routine blood sampling analysis, and coronary angiography examination by an experienced interventional cardiologist. The Table summarizes the number of eyes included, as well as patient demographics and comorbidities.

**Table.** Patient Numbers, Demography, and Comorbidities Per Severity Group

	Ordinal			Binary	
	Severity Group			Severity Group	
	0 (GS, 0–3)	1 (GS, 4–31)	2 (GS, >31)	0 (GS, 0–3)	1 (GS, >14)
Patients, *n*	73	86	53	73	88
Eyes (OD, OS), *n*	62, 54	68, 63	39, 45	62, 54	68, 72
Age (y), median (IQR)	58 (15)	60 (15)	62 (11)	58 (15)	61.5 (11)
Gender (male, female), *n*	36, 37	58, 28	41, 12	**36,** 37^*^	**69,** 19^*^
GS, median (IQR)	0 (0)	23 (7.5)	59 (29)	0 (0)	40.5 (39)
Arterial hypertension, *n* (%)	56 (76)	68 (74)	43 (81)	56 (76)	69 (78)
Diabetes mellitus, *n* (%)	14 (19)	22 (25)	17 (32)	14 (32)	26 (29)
Hyperlipidemia, *n* (%)	55 (75)	73 (84)	45 (84)	55 (75)	72 (81)
Family history of CAD, *n* (%)	35 (47)	33 (38)	27 (50)	35 (47)	43 (49)
Smoking, *n* (%)	35 (47)	45 (52)	31 (58)	35 (47)	47 (53)
Low-density lipoprotein (mg/dL), median (IQR)	**91.5 (61)** ^*^	**86 (46)** ^*^	**72.5 (49.7)** ^*^	**91.5 (61)** ^*^	**83 (51.5)** ^*^
HbA1c, median (IQR)	5.7 (0.5)	5.7 (0.62)	5.9 (0.8)	**5.7 (0.5)** ^*^	**5.8 (0.8)** ^*^
Creatinine (mg/dL), median (IQR)	0.84 (0.23)	0.84 (0.15)	0.88 (0.24)	0.84 (0.23)	0.86 (0.18)

Significance was tested using χ^2^ or Kruskal–Wallis test where applicable.

* Bold font Indicates *P* < 0.05 after Bonferroni correction.

We utilized the ZEISS PLEX Elite 9000 (Carl Zeiss Meditec, Jena, Germany) for retinal vascular image acquisition. This system employs a swept-source tunable laser with a center wavelength between 1040 nm and 1070 nm and a scan speed of up to 200 kHz A-scan rate. Volume scans of 6 × 6 mm (nominal size), centered on the macula, were acquired. From the resulting scans, en face images of the superficial capillary plexus were utilized. As the deep capillary plexus (DCP) is often prone to projection artifacts, resulting in unclear DCP FAZ borders, we decided not to include it in our analysis.^22^^–^^24^

### Gensini Score

In order to quantify the CAD severity, we applied an adjusted version of the established Gensini score (GS), which is described in detail in the original work by Gensini.^25^ In brief, the GS quantifies CAD based on the location and degree of stenosis. In addition to the traditional GS, we also took stenosis in the Ramus internus into account and assigned it the factor 1. Binary CAD severity groups were defined as follows: 0, GS 0 to 3; 1, GS > 14. The aim was to exclude patients with mere coronary sclerosis, not fitting the binary group definition of no stenosis versus significant CAD. For the three groups, the split was as follows: 0, GS 0 to 3; 1, GS 4 to 31; 2, GS > 31, where the last group represents patients with at least one complete stenosis (100%) or equivalent severity scores.

### FAZ Manual Annotation

Only images with excellent quality, where unambiguous annotation was possible, were assessed (Supplementary Table S2). We aimed to establish a standardized annotation procedure ensuring a consistent and repeatable FAZ assessment. Annotation of the FAZ was conducted manually using QuPath.^26^ One retinal expert (NJ) annotated the entire set and a second (SR) the first 300 images to prevent selection bias. Gray values belonging to the FAZ interior were inspected, and the highest gray value not representing an outlier was defined as border threshold. Moreover, defects in the FAZ border smaller than 2.5 pixels (∼14.65 µm) were not classified as significant alterations, yet they counted as part of the FAZ border (Supplementary Fig. S1).

### Image Analysis and FAZ Biomarkers

**Figure 1.**
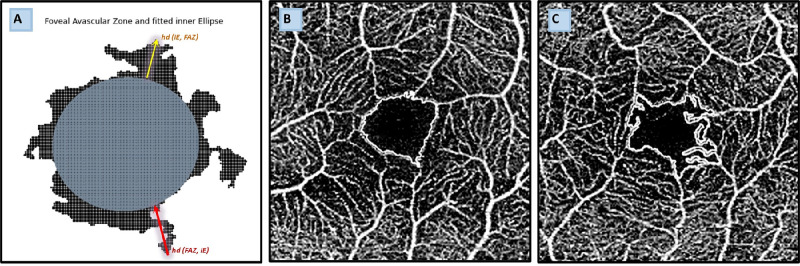
(**A**) Illustration of FAZ shape and the iE. The *red arrow* represents the FAZ to iE HD, and the *yellow arrow* represents the iE to FAZ HD. These show the largest smallest deviations of the two shapes. A point-cloud representation of the shapes is used for calculation of the CD, which can be interpreted as a more average discrepancy. Further calculation details for both metrics can be found in the Supplementary Materials. (**B**) A qualitative example of a FAZ in a healthy patient. (**C**) An irregular FAZ due to capillary drop-out in a patient with severe CAD.

The rationale behind our new biomarkers was the disrupted and irregular appearance of the FAZ in CAD patients compared to the round and solid FAZ seen in healthy individuals. This disruption may be attributed to capillary dropout from endothelial dysfunction or vascular remodeling.^27^ To objectively quantify these changes, we fitted an inner ellipse (iE) to approximate the original and healthy FAZ before capillary dropout. Subsequently, we calculated the absolute difference in the inner ellipse (diff iE) and used two well-known distance metrics, Hausdorff distance (HD) and Chamfer distance (CD), to quantify the deviation from the patient's FAZ and the iE (Fig. 1).

An algorithm was developed to approximate the largest inner ellipse. Defining the iE was achieved by utilizing an optimization with constraints, under the assumption that the geometrical center of the FAZ is inside the iE. The HD was calculated using Scipy.^28^ Considering two distinct shapes, *X* and *Y*, the HD is defined as the maximum distance from a point on the perimeter *X* to the nearest point on the perimeter of *Y* and vice versa. It emphasizes the largest deviation between two shapes, prioritizing the most significant discrepancy; subsequently, the greater distance is selected. For assessing the average distance between each point of the iE and the respective nearest points of the FAZ, the CD was computed. For shapes represented as point clouds, the CD is calculated as the average of the squared distances between a point from one set and its closest point in the other set. The calculations were conducted using a modified version of a PyTorch based implementation published by Ravi et al.^29^

By comparing the novel metrics against existing metrics, we aimed to evaluate the potential enhancements they offer over traditional FAZ biomarkers in the assessment of CAD. The following metrics were also computed: *area* (µm^2^); *circularity*, 4π × (area/perimeter^2^); *acircularity*, (FAZ perimeter)/(perimeter circle with equal area); *roundness*, (4 × area)/(π × major axis^2^); *solidity*, (FAZ area)/(convex hull area); and *convexity*, (FAZ perimeter)/(convex hull perimeter).

### Data Analysis

All parameters and analyzes were computed using Python and R (R Foundation for Statistical Computing, Vienna, Austria). Data normality and variance homogeneity were assessed using Shapiro–Wilk and the Levene's test. Subsequently, either one-way analysis of variance (ANOVA) with the post hoc Tukey's honestly significant difference method was conducted or, alternatively, a Kruskal–Wallis and Dunn's test with Holm's correction was carried out. Given the zero-inflated nature of our data (healthy patients with GS = 0) and the multiple violations of linear model assumptions, we selected a zero-inflated Poisson model to assess effect sizes while correcting for age, sex, and presence of diabetes mellitus. Separate models for left and right eyes were fitted. Additionally, to assess the predictive ability, binary logistic regression and ordinal (immediate threshold–based) logistic regression models were calculated considering only the left eyes.^30^ The above-defined severity groups were chosen as dependent variables. The synthetic minority oversampling technique (SMOTE) was performed for data oversampling for imbalanced datasets.^31^ Independent variables were age, sex (= baseline model), and one interchangeable FAZ metric. All evaluation metrics were averaged after performing stratified fivefold cross-validation. A post hoc analysis revealed dilatative cardiomyopathy, alcohol abuse, and aortic valve stenosis as confounders for FAZ irregularity, leading to the exclusion of 13 patients (22 eyes). Further information and the comprehensive results without post hoc exclusions are detailed in the Supplementary Materials. The Dice coefficient, Jaccard index, and Bland–Altmann plots were utilized for intergrader variability evaluation. All *P* values were adjusted for multiple testing and parameters using the Bonferroni correction.

## Results

**Figure 2.**
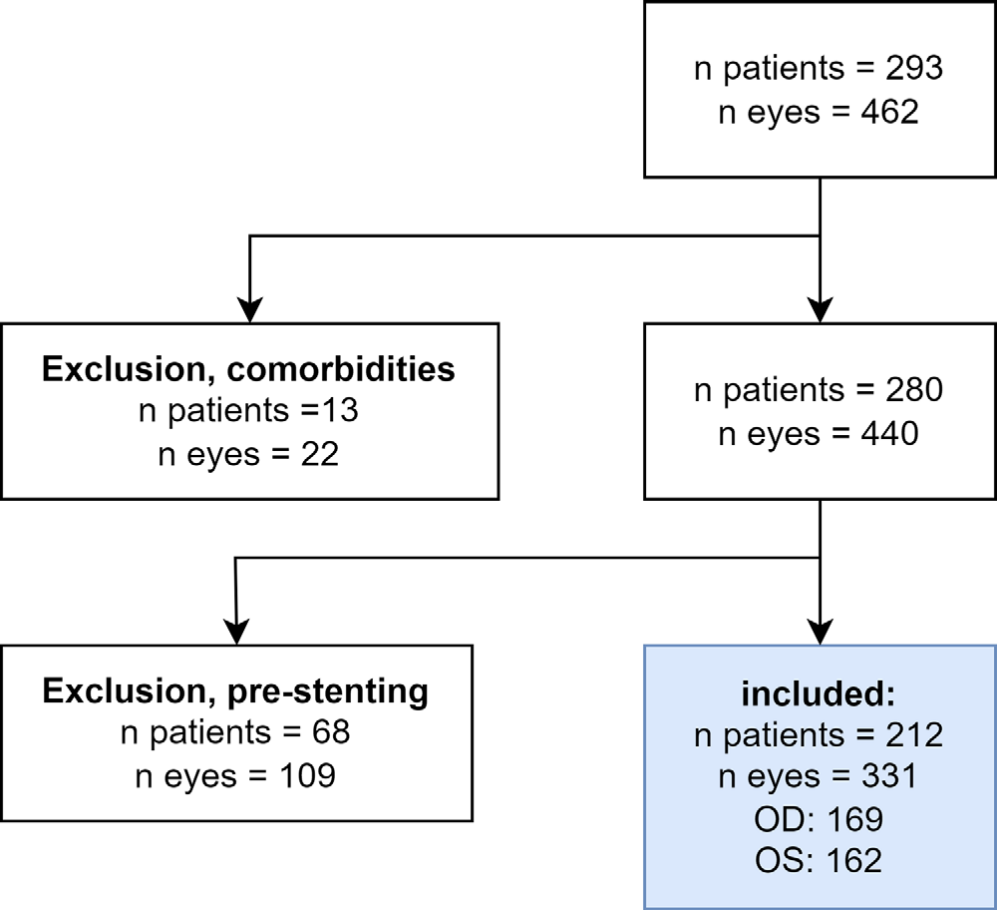
Flowchart depicting the exclusion process, resulting in a total number of 212 patients and 331 eyes. Sixty-eight patients (110 eyes) were excluded due to previous coronary stenting, and 13 patients (22 eyes) were excluded post hoc due to dilatative cardiomyopathy and alcohol abuse.

This study included 212 patients (331 eyes) who underwent coronary angiography (Fig. 2). Demographic and comorbidity profiles can be found in the Table.

### CAD Identification and Severity Stratification

**Figure 3.**
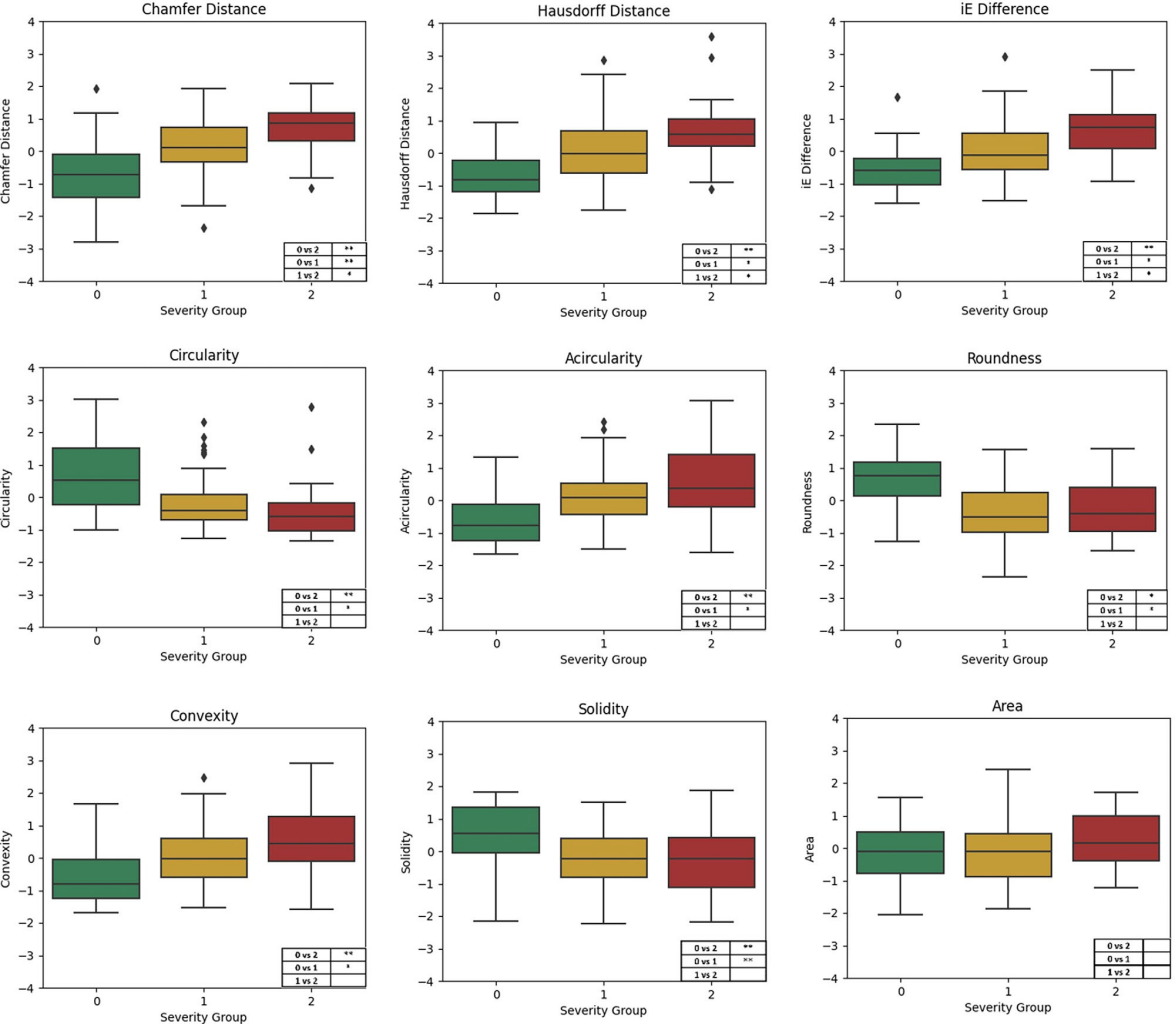
Boxplots depicting each FAZ parameter per CAD severity group in the left eye. For better interpretability, the parameters were *z*-standardized for the illustration. The table in the right corner of each plot shows the significance for the differences of each group combination tested with one-way ANOVA or Kruskal–Wallis test where applicable. Bonferroni-corrected significance is indicated as *P < 0.05; **P < 0.001.

Our results obtained in the left eye of study individuals demonstrated significant effects for all parameters except FAZ area. Conversely, the right eye showed no significant inflate parameter, suggesting that FAZ metrics of the right eye might in general not reliably indicate CAD absence (Supplementary Table S1). These findings are supported by the analysis of the differences across three CAD severity groups. Only the CD, HD, and diff iE of the left eye showed significance across all group combinations, indicating their superior potential in CAD severity assessment compared to other parameters (Fig. 3). Noteworthy, in the right eye no parameter showed a significant difference between groups 0 and 1 (Supplementary Table S2).

### Predictive Ability

**Figure 4.**
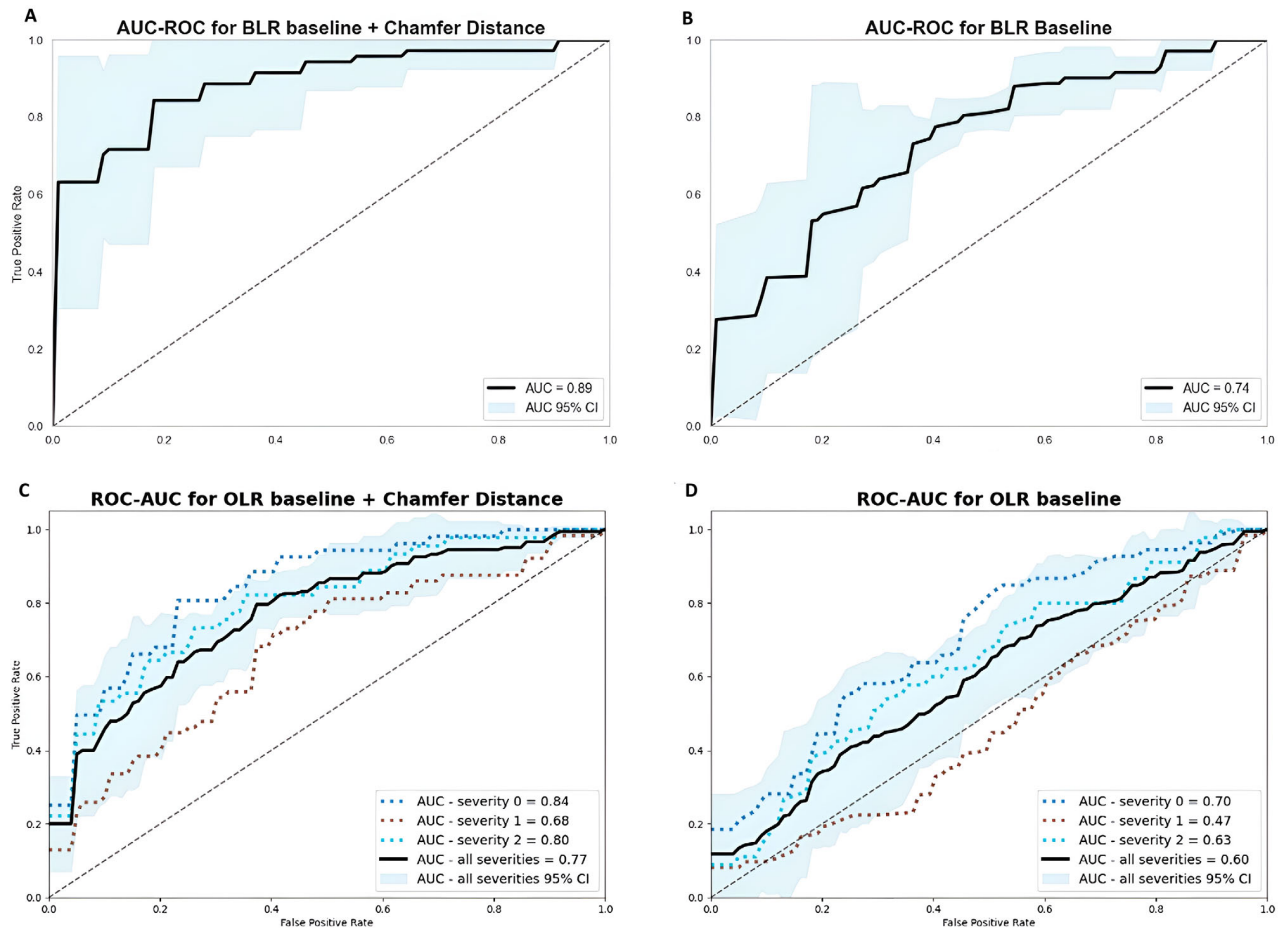
(**A**) AUC for the binary linear regression (BLR) model utilizing our best performing parameter (CD) in addition to age and sex, achieving an AUC of 0.89 (95% CI, 0.84–0.95) in discerning CAD and non-CAD patients. (**B**) The BLR baseline model with an AUC of 0.74 (95% CI, 0.64–0.81), not including any FAZ parameter but only age and sex. (**C**) AUC for the ordinal linear regression (OLR) model, discerning three CAD severity stages with an AUC of 0.77 (95% CI, 0.72–0.84). (**D**) In contrast, the OLR baseline model reached only 0.60 (95% CI, 0.53–0.65).

We explored the predictive ability of all parameters using binary and ordinal logistic regression models. CD and HD excelled in both models (Fig. 4). In the binary model, CD had the highest area under the receiver operating characteristic (ROC) curve (AUC) with a value of 0.891 (95% confidence interval [CI], 0.84–0.95), indicating superior ability for CAD discrimination, followed closely by HD with an AUC of 0.886 (95% CI, 0.84–0.94). In the ordinal logistic regression model, CD and HD again demonstrated the highest effectiveness in severity classification with AUCs of 0.769 (95% CI, 0.72–0.84) and 0.762 (95% CI, 0.72–0.83), respectively. These metrics further demonstrated significant improvements in F1 scores, accuracy, precision, and recall. Other parameters also showed good predictive capability, but at a less impactful level. Area and solidity were the least effective parameters (Supplementary Table S3).

### Intergrader Reliability

Intergrader reliability was high with a mean ± SD Dice coefficient of 0.926 ± 0.044 and a mean Jaccard index of 0.86 ± 0.1 (Bland–Altman plots for area, CD, and HD can be found in Supplementary Figures S3–S5). The area indicated a bias of –0.015 mm^2^, with limits of agreement (LoA) of –0.113 mm^2^ and 0.081 mm^2^. For the CD, a bias of –0.23, with LoA of –3.27 and 2.80, was observed. The data points are predominantly randomly distributed within the LoA, with minimal outliers. For the HD, the difference between the gradings became more pronounced as the scale increased (bias, –0.20e8; LoA, –0.21e9 and 0.17e9).

## Discussion

In this study, we aimed to evaluate the diagnostic value of the FAZ morphology on OCT-A as a retinal correlate for CAD. Our analyses revealed several important key findings.

1. The novel metrics (CD, HD, and diff iE) were the only parameters demonstrating significant differences across all three CAD severity groups.
2. CD and the HD showed the highest predictive ability for CAD identification and severity classification.
3. All metrics, except area, showed significant associations with the GS in the left eye.
4. Our standardized annotation process yielded high intergrader reliability.

Previous research has already hypothesized that a close relationship between ocular and systemic circulation exists. For example, fundus photography-based analyses have demonstrated that specific changes such as arterial narrowing and vessel caliber alterations can be found in patients with CAD.^32^^–^^34^ With the emergence of OCT-A, the field of retinal vascular imaging has been revolutionized, allowing for a more detailed assessment of the microvasculature.^35^ To analyze the resulting images, various standard parameters such as vessel density or fractal dimension have already been evaluated in cohorts similar to ours.^8^^,^^9^^,^^36^ The consensus from these studies strengthens the hypothesis that there is a pathognomonic association between retinal and cardiac circulation. However, the assessment of vessel density alone might be an unspecific biomarker, as it is altered in numerous retinal and systemic diseases, which raises doubts about their exclusive association with cardiac circulation.^17^^,^^37^^,^^38^ The lack of specificity points out the need for more reliable OCT-A biomarkers, whereby the combination of multiple features might offer the most comprehensive insight. In OCT-A–based studies focusing on CAD patients, further investigations to understand its diagnostic value are clearly needed.

Within this study, we assessed several FAZ shape descriptors. CD and HD demonstrated superior performance in binary and ordinal models, notably standing out in the ordinal model with significant distinction. Models incorporating the FAZ area seemed to exhibit limited performance compared to models utilizing other FAZ metrics. Additionally, CD, HD, diff iE, and perimeter were the only factors to show significant differences across all three severity groups. The intermediate CAD severity group, ranging from GS 3 to GS 31, is particularly interesting, as it included patients with coronary sclerosis and those with significant stenosis, leaving out patients with severe CAD. This subgroup potentially represents the optimal target group for screening (i.e., patients with subclinical incipient CAD). Naturally, this is also the most challenging condition to predict, as illustrated in Figures 4C and 4D. Although there is certainly room for refinement in distinguishing severity stages, our findings underscore the potential of retinal imaging as a non-invasive CAD screening tool. It is crucial to highlight that our results are grounded on single FAZ parameters. Therefore, our objective was not to fit high-performing prediction models, but rather to evaluate the predictive power of FAZ metrics in the realm of CAD risk stratification. Furthermore, along with other retinal metrics, the newly identified biomarkers could possibly serve as additional indicators within other risk stratification models for CAD. Existing research has already demonstrated a correlation between the American Heart Association risk or the Global Registry of Acute Coronary Events score and retinal vessel density, highlighting the potential of such integrations and representing a promising opportunity for future research.^8^

Interestingly, we observed that there was a varying degree of CAD-associated alterations in the retina, resulting in differing diagnostic capabilities between the left and right eye. This asymmetry might be explained by the increased hemodynamic stress and intimal damage of the left carotid artery due to its direct branching from the aorta.^39^^,^^40^ As the ophthalmic artery branches off the internal carotid artery, the left eye may be more prone to damage associated with cardiovascular diseases, because vascular damage potentially also influences the branches of the carotid artery and thus the tissues they supply. Although further validation in other patient cohorts is necessary, these findings underscore the importance of considering both eyes in patients with CVD and acknowledging that they may not be equally affected.

The FAZ area per se exhibited neither significant effect nor difference, which is consistent with findings from other studies.^8^^,^^12^^,^^13^ The FAZ area is subject to considerable variability due to ocular properties, such as axial length, along with demographic factors, such as age or sex.^23^^,^^41^^,^^42^ However, the CD, which is an averaged and relative metric, is not overly dependent on the absolute FAZ size and should therefore be robust against ocular magnification biases that affect measurements of the FAZ area. Moreover, in general it seems that parameters that focus on the irregularity and morphology instead of the absolute FAZ size tend to be more robust.^23^^,^^41^ For example, in other studies, circularity, which is also a relative FAZ metric, demonstrated no correlation with age, suggesting that it is less influenced by demographic factors.^11^ Although parameters such as circularity, acircularity, roundness, and solidity have already been assessed in other diseases, their application to CAD patients represents an innovative approach.^24^^,^^43^

Although FAZ annotation in healthy subjects usually demonstrates high reliability and repeatability, it may be more challenging to segment the FAZ consistently when it displays pronounced irregularities, as seen in diabetic retinopathy or retinal vein occlusion.^14^^,^^21^^,^^41^ One crucial factor influencing FAZ variability is the annotation process.^44^ Therefore, it seems essential to describe and define objective and quantifiable criteria to ensure the repeatability of the methodology. Using our previously described annotation thresholds, we managed to achieve excellent intergrader reliability, despite an unhealthy patient cohort that presented mostly with notably irregular FAZs.^45^ Our intergrader reliability aligned well with the reliability values found in studies on healthy eyes, substantially surpassing studies involving diseased eyes where the ICC ranges around 0.62.^14^

In the last decades, remarkable advancements have been made in coronary vessel assessment techniques, such as cardiac magnetic resonance, positron emission tomography, and cardiac computed tomography angiography. However, these methods are predominantly suitable for patients intended for coronary angiography rather than for screening purposes in asymptomatic patients.^46^^,^^47^ A further area of discussion is coronary microvascular dysfunction, which can be present without alteration in large coronary arteries. It is postulated that retinal microvasculature could serve as a surrogate for these cardiac microvessels, as they both have similar dimensions and anatomical architecture and are therefore hypothesized to undergo similar pathophysiological processes.^5^^,^^46^ Hence, developing non-invasive screening and diagnostic tools based on the retinal microvasculature holds great promise at a community-based level. This could be particularly beneficial for specific population subgroups for whom common risk assessment tools may not be well suited.^5^

It is assumed that the concept of endothelial dysfunction might be the underlying pathophysiology connecting the retinal and cardiac microvasculature.^48^^,^^49^ It is both a cause and a result of various pathophysiological processes, leading to long-term damage in microvascular end-organs such as the eye and heart.^50^ Microvascular dysfunction and damage in peripheral beds is known to reflect damage in visceral beds, validating the rationale for using the eye to detect systemic diseases.^51^^,^^52^ Existing literature demonstrates that endothelial dysfunction and oxidative stress seem to play a major role in atherosclerosis development and manifest a strong relationship with CAD and the risk of cardiovascular events.^53^^,^^54^ At the level of the retina, endothelial cell damage and pericyte loss can induce arterial narrowing and as a consequence a clinically significant impairment in blood flow.^55^ Given this context and considering the functionality of OCT-A, it is possible that vessels with absent or subthreshold flow are not visually displayed in the resulting images.^10^^,^^11^ We assume that these developments result in capillary dropout and a reduction in vessel density leading to the observed FAZ alterations.^27^^,^^56^ Similar pathophysiological processes are observed in diabetic patients, where patients display microvascular alterations such as loss of circularity and expansion of the FAZ, even without the presence of diabetic retinopathy.^6^^,^^14^^,^^43^^,^^55^ These findings underline the importance of adjusting for diabetes mellitus when assessing the influence of CVD on the retinal microvasculature.

Although this study has several strengths, it is essential to outline its limitations, as well. First, we relied on the manufacturer's automatic slab segmentation. Although, the algorithm performs well in general, and no significant flaws were detected during inspection, subtle errors cannot be ruled out. Moreover, lateral measurements reported in millimeters or micrometers were not rescaled based on axial length. This could lead to inaccuracies in the reported dimensions.^42^^,^^57^^,^^58^ Further, our study included a control group of patients without CAD, but they did have comorbidities such as arterial hypertension. However, these comorbidities showed a balanced distribution across the control group and the CAD groups, which should neutralize potential biases by reflecting changes that would likely influence all groups equally. Moreover, the participants in this study reflect typical clinical presentations of CAD patients, who scarcely ever are without any comorbidities. Moreover, the requirement of high-quality FAZ images in OCTA for reliable assessment may lead to the exclusion of numerous images. Finally, no cause and effect can be concluded from our findings, as correlation does not imply causality.

In summary, we conducted a comprehensive assessment of FAZ shape descriptors that to our knowledge have never been examined before in CAD. Moreover, we developed three innovative biomarkers based on the concept of an inner ellipse representing the original, healthy FAZ before capillary dropout. These novel metrics showed significant associations and differences across all CAD severity groups. A most relevant result was the ability to discern CAD and non-CAD patients and moreover distinguish between three severity stages utilizing the CD. Our findings emphasize that the FAZ does indeed have significant diagnostic potential in CAD, challenging previous skepticism.^59^^,^^60^ Additional studies using diverse patient cohorts will be necessary to examine the CAD specificity of the new metrics and assess their diagnostic potential in related systemic diseases.

## Supplementary Material

Supplement 1
