# Supplementary material for: Severity Stratification of Coronary Artery Disease Using Novel Inner Ellipse-Based Foveal Avascular Zone Biomarkers
Source: Invest Ophthalmol Vis Sci. 2024 Oct 9;65(12):15. doi: 10.1167/iovs.65.12.15 (PMC11469242; doi:10.1167/iovs.65.12.15)
Supplement: Supplement 1 [file iovs-65-12-15_s001.pdf]

Supplement

Image Quality and FAZ Annotation

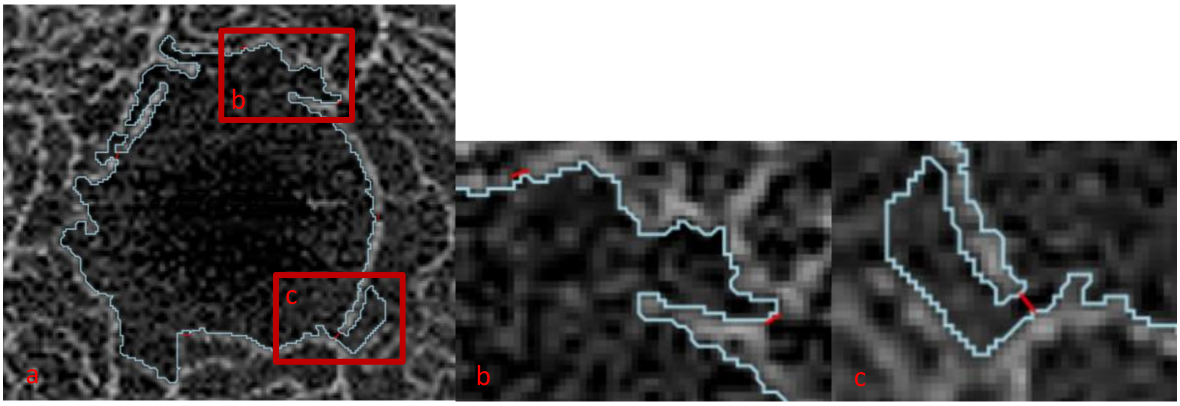

Figure S1: *a) exemplary full size manual FAZ Annotation; b) Example of non-significant holes in the FAZ border (pixels with grey value below the defined border-threshold) that are <2.5 pixel (~14.65 µm). c) Example of a significant hole in the FAZ border (>2.5 pixel)*

	Grading	Grading Description
**1a**	Excellent	The FAZ is in optimal condition without any artifacts or impairments.
**1b**	Small artifacts outside the FAZ region	The FAZ is unaffected by artifacts, although there may be minor artifacts present elsewhere in the image
**1c**	Small artifacts in the FAZ region	There are small artifacts present within the FAZ region, but they do not significantly affect the evaluation of the FAZ
**2**	FAZ impaired but potentially acceptable	The FAZ shows some impairment, such as blurred boundaries or partial obstruction, but the overall quality may still be acceptable for analysis. Need for caution in analyzing.
**3**	Poor Quality	The FAZ is significantly impaired and unsuitable for reliable assessment and analysis.

Table S1: *Quality Grading criteria for image inclusion*

A quality score of 1 (a-c) comprises images with excellent quality or only small artifacts not affecting the evaluation of the FAZ significantly. Images with grading 2 show some impairment (marginally blurred boundaries, partial obstruction) but the overall quality may still be acceptable, although there is need for caution in analyzing. Grading 3 means that the FAZ is significantly impaired and unsuitable for reliable assessment. Grade 3 images were excluded. Grade 2 images were revised a second time and excluded if unambiguous annotation was not possible.

Gensini Score – Extended Explanation

In order to quantify the CAD severity, we applied an adjusted version of the established Gensini Score (GS), which is described in detail in the original work by Gensini et.al. [25] The Gensini score quantifies CAD, based on location and degree of coronary stenosis in percent. Additionally, to the traditional GS we took stenoses in the Ramus internus into account and assigned them the factor 1. Binary CAD severity groups were defined as follows: 0: GS 0-3; 1: GS >14, aiming to exclude patients with mere coronary sclerosis, not fitting the binary group definition of no stenoses vs. significant CAD. By using this certain split, we aim to focus on patients with clear-cut cases of CAD versus those without. Specifically, we aimed to exclude patients with coronary sclerosis (incipient CAD), as these patients are neither completely healthy and free of stenoses, nor do they have a clear CAD diagnosis. For a more advanced analysis and in order to leverage the ability of CAD severity staging using the Gensini Score we decided to perform analyzes on a three-group-split in addition. The main goal of this split was to evaluate if even incipient/moderate CAD stages can be identified and moreover to assess if the retinal microvasculature differs in patients with incipient/moderate CAD compared to severe/complex CAD. For the three group distinction the split was 0: GS 0-3, 1: GS 4-31 and 2: GS >31, where the latter group threshold represents patients with at least one complete stenosis (100%) or equivalently complex severity scores.

FAZ Biomarkers- Calculation Details

The circularity quantifies how close the FAZ shape is to a perfect circle, it embodies the FAZ area as well as the FAZ perimeter, whereby the value 1 indicates a perfect circular shape. Acircularity is a similar measure, though focused on the perimeter relations. The convex hull is the smallest convex polygon that encloses the FAZ. By calculating the ratio between the FAZ area and the convex hull area, information about the FAZ solidity and integrity can be provided. On the other hand, the convexity, takes the ratio of the perimeters into account instead.

Defining the inscribed ellipse is achieved by utilizing an optimization with constraints, under the assumption that the geometrical center of the FAZ is inside the iE. The ellipse is represented as a unit sphere under affine transformation, a quadratic form with the symmetric matrix $f(A, b, x) = \hat{x}^T A \hat{x} - 1 = 0$, where b is the translation and $\hat{x} = x - b$. Subsequently, we aim to recover the parameters A and b by minimizing the objective function defined as mean of the square of the quadratic form f calculated at all FAZ boundary locations x under the constrain that all of the points x are outside of the ellipse. ($\hat{x}^T A \hat{x} - 1 \geq 0$) To solve this optimization problem, we employed the "SLSQP" method described by Kraft et. al. [45]

Considering two distinct shapes, X and Y, the Hausdorff Distance is defined as the maximum distance from a point on the perimeter X to the nearest point on the perimeter of Y and vice versa. Subsequently, the greater distance is selected. Mathematically it is defined as:

$$HD(X, Y) = \max\,[hd(X, Y),\ hd(Y, X)]$$

Given two sets of points A and B, representing distinct shapes, the CD formula presents as:

$$CD(A, B) = \frac{1}{|A|} \sum_{a \in A} \min_{b \in B} ||a - b||^2 + \frac{1}{|B|} \sum_{b \in B} \min_{a \in A} ||a - b||^2$$

Where |A| and |B| symbolize the number of points per set and ||a-b|| depicts the Euclidean distance between two points. The calculations were conducted using a modified version of a PyTorch based implementation published by Ravi et.al. [46]

Detailed Results and Performance Metrics

OS

parameter	*coefficent*	*CI 95%*	*z - value*	*p-value*
CD	**0.48**	**0.443, 0.516**	**25.783**	**<0.001***
inf- CD	**-1.223**	**-1.692, -0.754**	**-5.115**	**<0.001***
HD	**1.408**	**1.290, 1.525**	**23.521**	**<0.001***
inf- HD	**-3.779**	**-5.243, -2.315**	**-5.061**	**<0.001***
iE Diff.	**1.075**	**0.981, 1.168**	**22.514**	**<0.001***
inf- iE Diff.	**-2.728**	**-3.888, -1.567**	**-4.608**	**<0.001***
Circularity	**-3.076**	**-3.483, -2.669**	**-14.814**	**<0.001***
inf- Circularity	**8.838**	**5.276, 12.399**	**4.864**	**<0.001***
Acircularity	**0.108**	**0.092, 0.123**	**13.86**	**<0.001***
inf- Acircularity	**-0.6**	**-0.869, -0.331**	**-4.37**	**<0.001***
Solidity	**-1.13**	**-1.607, -0.651**	**-4.631**	**<0.001***
inf- Solidity	**12.281**	**5.934, 18.626**	**3.793**	**0.003***
Covexity	**0.466**	**0.400, 0.532**	**13.866**	**<0.001***
inf- Covexity	**-2.525**	**-3.688, -1.361**	**-4.254**	**<0.001***
Area	**0.679**	**0.572, 0.785**	**12.452**	**<0.001***
inf- Area	-1.377	-2.522, -0.232	-2.358	0.331
Roundness	**-1.056**	**-1.404, -0.707**	**-5.941**	**<0.001***
inf- Roundness	**8.413**	**4.166, 12.659**	**3.883**	**0.002***

OD

parameter	*coefficent*	*CI 95%*	*z - value*	*p-value*
CD	**0.311**	**0.280, 0.341**	**20.091**	**<0.001***
inf- CD	-0.36	-0.719, 9.04e-05	-1.959	0.901
HD	**0.94**	**0.841, 1.039**	**18.616**	**<0.001***
inf- HD	-1.223	-2.384, -0.061	-2.064	0.703
iE Diff.	**0.761**	**0.679, 0.842**	**18.255**	**<0.001***
inf- iE Diff.	-0.64	-1.572, 0.292	-1.345	1.0
Circularity	**-3.191**	**-3.551, -2.830**	**-17.356**	**<0.001***
inf- Circularity	1.491	-1.684, 4.667	0.92	1.0
Acircularity	**0.143**	**0.126, 0.158**	**17.413**	**<0.001***
inf- Acircularity	-0.165	-0.366, 0.036	-1.605	1.0
Solidity	**-3.828**	**-4.306, -3.349**	**-15.686**	**<0.001***
inf- Solidity	3.001	-2.633, 8.635	1.044	1.0
Covexity	**0.613**	**0.543, 0.682**	**17.218**	**<0.001***
inf- Covexity	-0.676	-1.549, 0.197	-1.516	1.0
Area	**0.434**	**0.331, 0.536**	**8.297**	**<0.001***
inf- Area	-0.126	-1.208, 0.956	-0.228	1.0
Roundness	**-2.5**	**-2.838, -2.161**	**-14.463**	**<0.001***
inf- Roundness	1.807	-2.118, 5.733	0.902	1.0

Table S2: ***Statistics from the Zero-Inflated Poisson model*** *used to analyze data where zero outcomes (e.g. no coronary artery disease, GS=0) are common. It combines a Poisson count model with a logistic model for predicting the occurrence of zero counts. The variables without prefix show how FAZ parameters affect the Gensini score (=CAD severity) and the 'inflate' variables assess the chance of having a GS of 0 (=CAD identification). Additional independent variables were age, sex and DM presence. All p-values were adjusted using Bonferroni Correction.*

	OD			***OS***		
Severity Group	**0**	**1**	**2**	**0**	**1**	**2**
CD	12.66	12.73	13.53	12.04	12.92	13.68
HD	33.48	34.76	44.03	28.19	37.35	44.76
diff iE (µm²)	104215.68	108056.95	132260.49	89320.50	109225.84	140538.41
Circularity	0.24	0.23	0.16	0.30	0.20	0.16
Acircularity	6.96	7.19	8.57	6.27	7.67	8.71
Solidity	0.81	0.81	0.76	0.84	0.78	0.78
Roundness	0.59	0.59	0.53	0.63	0.54	0.54
Convexity	1.87	1.93	2.22	1.73	2.02	2.28
Area(µm²)	323059.02	319641.16	332782.63	312445.35	310951.49	353123.64

	**OD**			**OS**		
	**0 vs 2**	**0 vs 1**	**1 vs 2**	**0 vs 2**	**0 vs 1**	**1 vs 2**
**CD**	**0,87****	0,07	**0,80***	**1,64****	**0,88****	**0,76***
**HD**	**10,55****	1,29	**9,27***	**16,57****	**9,15****	**7,41***
**diff iE (µm²)**	**28044,81***	3841,27	**24203,54***	**51217,91****	**19905,34***	**31312,56****
**Circularity**	**-0,08****	0,00	**-0,08***	**-0,14****	**-0,11****	-0,04
**Acircularity**	**1,60****	0,23	**1,37***	**2,44****	**1,40****	1,04
**Solidity**	**-0,05***	0,00	-0,05	**-0,06****	**-0,06****	-0,01
**Roundness**	**-0,06****	0,00	**-0,06***	**-0,09****	**-0,09***	0,00
**Convexity**	**0,35****	0,05	0,30	**0,55****	**0,29***	0,26
**Area(µm²)**	9723,61	-3417,87	13141,48	40678,29	-1493,86	42172,15

Table S3: ***Mean values per severity groups for each eye in the and absolute differences discerned between the three severity groups****. The table employs ANOVA and Kruskal-Wallis (KW) tests to test significant differences between the groups, where **=p<0.001, *=p<0.05. Most notably, the newly introduced parameters—Chamfer Distance (CD), Hausdorff Distance (HD), and difference to inscribed ellipse (diff iE)—manifest significance across all three groups in the left eye. This suggests their potential in severity staging, contrasting with nearly all other parameters that fail to exhibit significance across all groups.*

BINARY LOGISTIC REGRESSION

	AUC-ROC (CI 95%)	***F1 Score***	***Accuracy***	***Precision***	***Recall***	***BIC***	***LL***	***LLR***	***LLR p***
Baseline (age, sex)	*0.738 (0.64, 0.81)*	*0.745*	*0.682*	*0.701*	*0.805*	*181.342*	*-83.217*	*-*	*-*
+ CD	0.891 (0.84, 0.95)	0.813	0.793	0.866	0.790	131.493	-55.807	54.818	1.3 e^{-13}
+ HD	0.886 (0.84, 0.94)	0.795	0.777	0.831	0.775	134.675	-57.398	51.637	6.6 e^{-13}
+ diff iE	0.839 (0.78, 0.93)	0.727	0.571	0.571	1.0	154.778	-67.450	31.533	1.9 e^{-08}
+ circ	0.828 (0.79, 0.92)	0.781	0.738	0.773	0.805	150.702	-65.411	35.610	2.4 e^{-09}
+ acirc	0.844 (0.79, 0.92)	0.757	0.737	0.822	0.721	150.025	-65.073	36.287	1.7 e^{-09}
+ solid	0.784 (0.75, 0.89)	0.756	0.698	0.724	0.805	163.573	-71.847	22.739	1.8 e^{-06}
+ convex	0.848 (0.78, 0.92)	0.807	0.785	0.857	0.777	153.644	-66.883	32.668	1.1 e^{-08}
+ round	0.812 (0.76, 0.90)	0.766	0.714	0.741	0.804	161.232	-70.676	25.081	5.5 e^{-07}
+ area	0.599 (0.51, 0.69)	0.727	0.571	0.571	1.0	181.894	-81.007	4.418	0.035

ORDINAL LOGISTIC REGRESSION

		AUC-ROC (CI 95%)	***F1 score***	***Accuracy***	***Precision***	***Recall***	***BIC***	***LL***	***LLR***	***LLR p***
Baseline (age, sex)	micro	0.607 (0.53, 0.65)	0.408	0.408	0.408	0.408	524.887	-254.812	-	-
	macro		0.38		0.417	0.440				
+ CD	micro	0.769 (0.72, 0.84)	0.612	0.612	0.612	0.612	333.862	-156.755	196.112	1.47 e^{-44}
	macro		0.611		0.607	0.625				
+ HD	micro	0.762 (0.72, 0.83)	0.587	0.587	0.587	0.587	350.574	-165.112	179.400	6.55 e^{-41}
	macro		0.580		0.584	0.600				
+ diff iE	micro	0.684 (0.63, 0.73)	0.407	0.407	0.407	0.407	379.399	-179.524	150.575	1.29 e^{-34}
	macro		0.274		0.292	0.368				
+ circ	micro	0.692 (0.64, 0.76)	0.463	0.463	0.463	0.463	450.430	-215.040	79.543	4.72 e^{-19}
	macro		0.454		0.456	0.486				
+ acirc	micro	0.734 (0.67, 0.79)	0.495	0.495	0.495	0.433	390.272	-184.960	139.702	3.09 e^{-32}
	macro		0.476		0.479	0.461				
+ solid	micro	0.632 (0.58, 0.70)	0.433	0.433	0.433	0.433	511.399	-245.524	18.575	1.63 e^{-05}
	macro		0.417		0.434	0.460				
+ convex	micro	0.723 (0.67, 0.80)	0.500	0.500	0.50	0.500	393.228	-186.438	136.746	1.37 e^{-31}
	macro		0.489		0.499	0.515				
+ round	micro	0.636 (0.59, 0.70)	0.408	0.408	0.408	0.408	493.315	-236.482	36.659	1.41 e^{-09}
	macro		0.387		0.373	0.434				
+ area	micro	0.544 (0.49, 0.62)	0.389	0.389	0.389	0.390	388.699	-184.174	141.276	1.4 e^{-32}
	macro		0.187		0.130	0.333				

Table S4: ***Deteiled comparison of the predictive ability of individual parameters withing the binary logistic regression model (BLR) and ordinal logistic regression model (OLR).*** *The baseline model incorporates only sex and age. FAZ parameters were added to the baseline model separately to evaluate their respective performances and significances. Log-Likelihood (LL), Log-Likelihood ratio (LLR), and its p value were calculated between the baseline model and each model with additional FAZ parameter. Notably, the CD consistently outperforms other parameters across all evaluation metrics for both BLR and OLR, indicating its predictive superiority. the CD performed the best in every single evaluation metric for both BLR and OR. CD=Chamfer Distance, HD=Hausdorff distance, convex = convexity, circ=circularity, peri=perimeter, round=roundness, diff= difference iE, solid=solidity*

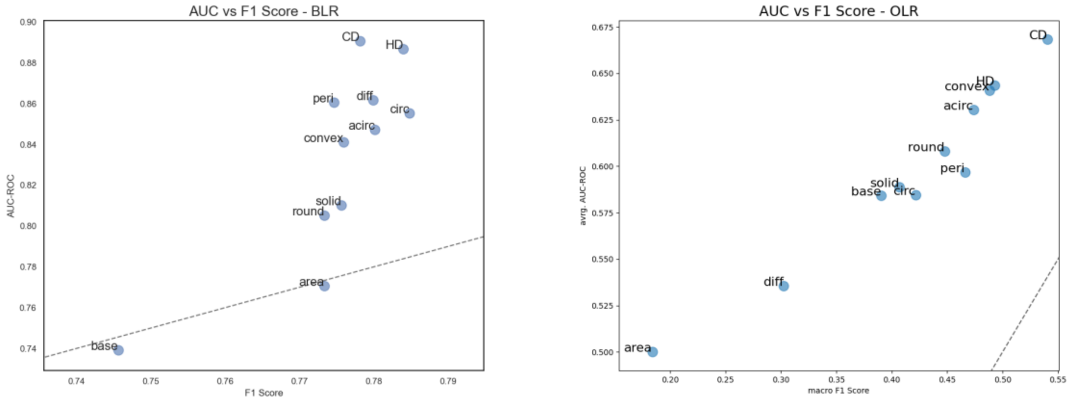

<u>Figure S2:</u> *Illustration of the performance of each the ordinal and binary logistic regression models using ROC-AUC and F1 Score as evaluative metrics. Macro versions of the AUC and F1 Score were employed for the OLR model. For each model one FAZ metric was set as additional parameter to the baseline model (base). CD=Chamfer Distance, HD=Hausdorff distance, convex = convexity, circ=circularity, peri=perimeter, round=roundness, diff= difference iE, solid=solidity*

Bland Altman Plots

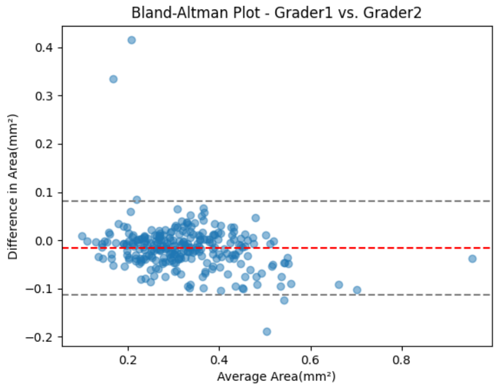

<u>Figure S3:</u> *Bland Altmann Plot showing the intergrader variability. A bias of -0.015 mm², with lower and upper LoA of -0.113 mm² and 0.081 mm² respectively were observed. Analyzing the outliers in the BA plot, poor image quality and low signal intensity were identified as sources of discrepancy. The choice of grey value border-thresholds may represent a further contributor to variation, with potential for standardization in future works.*

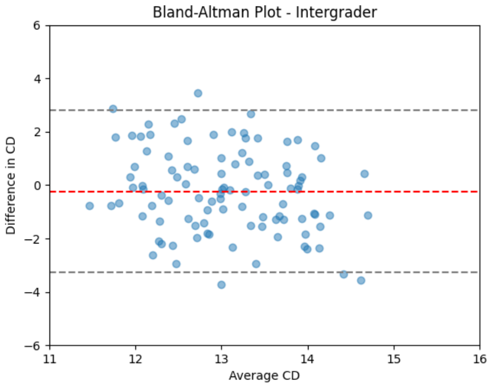

<u>Figure S4:</u> *Bland Altmann Plot showing the intergrader variability for the logarithmically transformed Chamfer Distance, which was used for all calculations. A bias of -0.23, with lower and upper LoA of -3.27 and 2.80 respectively were observed. The data points are predominantly within the LoA, with minimal outliers. Points are randomly distributed around the mean difference line.*

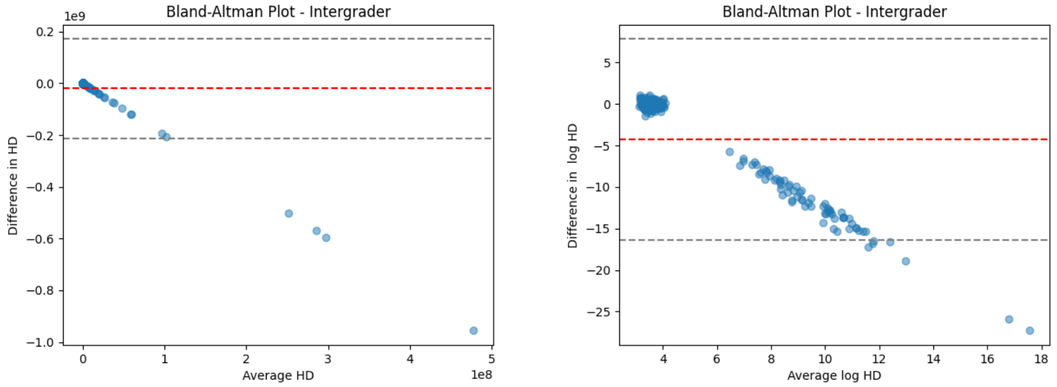

<u>Figure S5:</u> *Bland Altmann Plot showing the intergrader variability for the logarithmically transformed Chamfer Distance. A bias of -0.20e8, with lower and upper LoA -0.21e9 and 0.17e9 respectively were observed. For visualization purposes the right image depicts the BA plot with the logarithmically transformed HD. (LoA of -16.34 and 7.82, bias -0.23). It is clearly visible that the difference between the metrics becomes more pronounced as the scale increases.*

Results without post-hoc exclusions

While conducting a post-hoc analysis, aortic valve stenosis, dilatative cardiomyopathy and alcohol abuse were identified as potential confounders affecting FAZ irregularity. This resulted in the exclusion of 13 additional patients (22 eyes). Our primary aim was to investigate the associations between FAZ metrics and CAD without comprehensive adjustment of confounders, as a comprehensive multivariate approach would be beyond the scope of this study. Thereby, we also wanted to avoid the risk of biased interpretations from selectively adjusting only a few confounders that were present in a limited number of patients only. Moreover, some patients, such as those with severe aortic valve stenosis, typically do not undergo coronary angiography due to suspected CAD but as part of preoperative screening prior to procedures like aortic valve replacement. These patients are often classified as 'healthy' in terms of the Gensini Score because they often show no coronary stenoses. Their inclusion could therefore lead to skewed results due to their severe cardiological conditions that may alter the retinal microvasculature regardless of CAD. Hence, in order to keep a homogenous study population and ensure robust statistical analysis, these outliers were excluded. However, to also maintain transparency and provide a comprehensive view of our findings we included the complete results without post-hoc exclusions in the following.

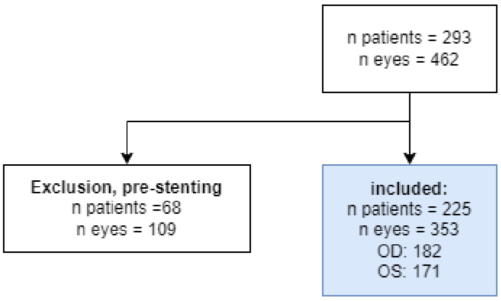

<u>Figure S6:</u> *Flowchart depicting the exclusion process without post-hoc exclusion, resulting in a total number of 225 patients and 353 eyes. 68 patients (109 eyes) were excluded due to previous coronary stenting.*

	ordinal			binary	
**Severity Group**	**0**	**1**	**2**	**0**	**1**
GS	(0-3)	(4 – 31)	(>31)	(0-3)	(>14)
**Patients (n)**	81	90	54	81	89
**n eyes (OD|OS)**	70|61	71|64	40|46	70|61	69|73
**median age (IQR)**	58 (14,7)	60 (15)	61 (11)	58 (14,7)	61 (11)
**sex (m|f)**	45|36	62|28	42|12	**45|36***	**70|19***
**median GS (IQR)**	0 (0)	12 (7,5)	56 (29)	0 (0)	40 (38)
**aHT (%)**	62(76)	72(80)	44(81)	62(76)	70(79)
**DM (%)**	17(21)	24(27)	17(31)	17(21)	26(29)
**HLP (%)**	59(73)	76(84)	46(85)	59(73)	73(82)
**FH (%)**	36(44)	36	27(50)	36(44)	43(48)
**Smoking (%)**	41(51)	49(54)	32(59)	41(51)	48(54)
**median LDL (IQR)**	**93 (55) ***	**86 (46) ***	**74 (50) ***	**97,16 (±36,97) ***	**86,14 (±36,97) ***
**median HbA1c (IQR)**	5,7 (0,5)	5,7 (0,6)	5,9 (0,8)	**5,7 (0,5) ***	**5,8 (0,8) ***
**median Crea (IQR)**	0,86 (0,2)	0,84 (0,1)	0,88 (0,2)	0,86 (0,2)	0,86 (0,2)

Table S5: *Patient numbers, demography, and comorbidities per severity group. Significance was tested using chi-square or Kruskal Wallis test where applicable. * indicates a p-value <0,05 after Bonferroni correction. GS=Gensini score, aHT=arterial hypertension, DM=Diabetes Mellitus, HLP=hyperlipidemia, FH=family history of CAD, LDL=low-density lipoprotein (mg/dl), Crea=serum creatinine (mg/dl)*

Group Means & SD

	OD			***OS***		
Severity Group	**0**	**1**	**2**	**0**	**1**	**2**
CD	12.61 (±1.08)	12.70 (±1.08)	13.53 (±1.08)	12.17 (±1.12)	12.93 (±1.12)	13.66 (±1.12)
HD	33.24 (±13.30)	34.42 (±13.30)	43.78 (±13.30)	29.47 (±12.26)	37.39 (±12.26)	44.60 (±12.26)
diff iE (μm²)	102488.49 (±46509.16)	106640.45 (±46509.16)	132295.83 (±46509.16)	88141.84 (±43018.28)	109857.59 (±43018.28)	139771.32 (±43018.28)
Circularity	0.23 (±0.11)	0.24 (±0.11)	0.15 (±0.11)	0.29 (±0.12)	0.20 (±0.12)	0.16 (±0.12)
Acircularity	7.01 (±1.94)	7.14 (±1.94)	8.63 (±1.94)	6.42 (±2.04)	7.65 (±2.04)	8.73 (±2.04)
Solidity	0.81 (±0.07)	0.81 (±0.07)	0.76 (±0.07)	0.83 (±0.07)	0.78 (±0.07)	0.77 (±0.07)
Roundness	0.59 (±0.09)	0.59 (±0.09)	0.53 (±0.09)	0.62 (±0.10)	0.54 (±0.10)	0.53 (±0.10)
Convexity	1.89 (±0.44)	1.91 (±0.44)	2.24 (±0.44)	1.75 (±0.47)	2.01 (±0.47)	2.28 (±0.47)
Area(μm²)	312712.58 (±104628.16)	316980.03 (±104628.16)	332124.82 (±104628.16)	302644.25 (±96469.27)	312401.85 (±96469.27)	351491.11 (±96469.27)

Group Differences

	OD			***OS***		
	**0 vs 2**	**0 vs 1**	**1 vs 2**	**0 vs 2**	**0 vs 1**	**1 vs 2**
**CD**	**0,915****	0,081	**0,834****	**1,493****	**0,763****	**0,73***
**HD**	**10,542***	1,18	**9,362***	**15,136****	**7,922***	**7,214***
**diff iE (μm²)**	29807,334	4151,957	**25655,377***	**51629,48****	**21715,749***	**29913,731***
**Circularity**	**-0,079***	0,002	**-0,081****	**-0,132****	**-0,091***	-0,041
**Acircularity**	**1,624****	0,129	**1,495***	**2,312****	**1,229***	1,083
**Solidity**	-0,051	0,001	**-0,052***	**-0,054****	**-0,045****	-0,009
**Roundness**	**-0,059***	0	**-0,059***	**-0,084***	**-0,078***	-0,006
**Convexity**	**0,353***	0,028	**0,325***	**0,529****	**0,261***	0,268
**Area(μm²)**	19412,243	4267,45	15144,793	48846,856	9757,599	39089,257

Table S6: *Mean values per severity groups for each eye and absolute differences discerned between the three severity groups. The table employs one-way ANOVA and Kruskal-Wallis (KW) tests to examine significant differences between the groups, where **=p<0.001, *=p<0.05. The p-values were corrected for multiple comparisons and the number of parameters tested. Most notably, the newly introduced parameters—Chamfer Distance (CD), Hausdorff Distance (HD), and difference to inscribed ellipse (diff iE)—manifest significance across all three groups in the left eye.*

BINARY LOGISTIC REGRESSION

	AUC-ROC	F1 Score	Accuracy	Precision	Recall	BIC	LL	LLR	LLR p
Baseline (age, sex)	0.707	0.699	0.657	0.663	0.596	195.309	-90.179	0.0	0.0
+ CD	0.852	0.731	0.753	0.786	0.638	151.767	-65.916	48.525	3.26 e-12
+ HD	0.841	0.741	0.746	0.765	0.622	151.617	-65.841	48.676	3.02 e-12
+ diff iE	0.823	0.732	0.545	0.804	0.653	165.315	-72.69	34.978	3.33 e-09
+ circ	0.782	0.727	0.679	0.738	0.616	164.16	-72.113	36.132	1.84 e-09
+ acirc	0.827	0.729	0.723	0.78	0.624	168.077	-74.071	32.215	1.38 e-08
+ solid	0.735	0.721	0.634	0.706	0.598	179.873	-79.969	20.42	6.22 e-06
+ convex	0.821	0.721	0.708	0.78	0.624	166.134	-73.1	34.159	5.08 e-09
+ round	0.749	0.717	0.657	0.726	0.608	178.595	-79.33	21.697	3.19 e-06
+ area	0.623	0.719	0.545	0.718	0.587	188.925	-84.495	11.368	0.0007

ORDINAL LOGISTIC REGRESSION

		AUC-ROC	F1 score	Accuracy	Precision	Recall	BIC	LL	LLR	LLR p
Baseline (age, sex)	micro	0.592	0,386	0,386	0,386	0,386	598.987	-291.781	-	-
	macro		0,345		0,345	0,412				
+ CD	micro	0.748	0.538	0,538	0.538	0.538	373.314	-176.374	230.815	3,96 e^{-52}
	macro		0.522		0.522	0,548				
+ HD	micro	0.748	0,527	0,527	0,527	0,527	394.619	-187.026	209.509	1,76 e^{-47}
	macro		0.508		0.508	0,539				
+ diff iE	micro	0.678	0,421	0,421	0,421	0,421	397.835	-188.634	206.294	8,84 e^{-47}
	macro		0.311		0.311	0,4				
+ circ	micro	0.683	0,45	0,45	0,45	0,45	495.714	-237.574	108.414	2,18 e^{-25}
	macro		0.427		0.427	0,465				
+ acirc	micro	0.716	0,474	0,474	0,474	0,474	425.343	-202.388	178.785	8,93 e^{-41}
	macro		0,447		0,447	0,482				
+ solid	micro	0.620	0,415	0,415	0,415	0,415	582.889	-281.161	21.24	4,05 e^{-06}
	macro		0.447		0.447	0,436				
+ convex	micro	0.716	0,48	0,48	0,48	0,48	428.441	-203.937	175.688	4,24 e^{-40}
	macro		0.379		0.379	0,492				
+ round	micro	0.628	0,427	0,427	0,427	0,427	550.053	-264.743	54.075	1,93 e^{-13}
	macro		0.427		0.427	0,443				
+ area	micro	0.550	0,374	0,374	0,374	0,374	*406.762*	-193.098	197.366	7,84 e^{-45}
	macro		0.453		0.453	0,333				

Table S7: *Detailed comparison of the predictive ability of individual parameters within the binary logistic regression model (BLR) and ordinal logistic regression model (OLR). BIC=bayesian information criterion, CD=Chamfer Distance, HD=Hausdorff distance, convex = convexity, circ=circularity, peri=perimeter, round=roundness, diff iE= difference to iE, solid=solidity*
